# Factors contributing to delays in accessing health facility-based maternal delivery services in Sierra Leone, 2018: A community-based cluster survey

**DOI:** 10.1371/journal.pone.0307179

**Published:** 2024-09-17

**Authors:** Gbessay Saffa, Charles Keimbe, Andrew Bangalie, Amara Alhaji Sheriff, Babah Jalloh, Doris Bah, Fatmata Bangura, Francis Tamba, Henry Bangura, Isha Sesay, Kassim Kamara, Sahr Gborie, Hale Teka, Eric Ikoona, Adel Hussein Elduma, Gebrekrstos Negash Gebru

**Affiliations:** 1 Ministry of Health, Sierra Leone; 2 Mekelle University, College of Health Sciences, Mekelle, Ethiopia; 3 Track Trust, SCIO, Scotland, United Kindom; 4 ICAP at Columbia University in Sierra Leone, Sierra Leone; 5 African Field Epidemiology Network, Field Epidemiology Training Program, Sierra Leone; University of Ottawa, CANADA

## Abstract

**Background:**

With a Maternal Mortality Ratio (MMR) of 516 deaths per 100,000 live-births, Sierra Leone hosts one of the highest maternal mortalities in globally. National data indicates that over 98% of maternal deaths are related to delays in accessing obstetric services. This study sought to examine factors contributing to delays in accessing maternal delivery services as perceived by women in Sierra Leone.

**Methods:**

We conducted a community-based survey among women who delivered from May 1, 2017 to June 30, 2018, in four districts of Sierra Leone. Delay one was defined as perceived delays in deciding to seek facility-based delivery. Delay two was defined as perceived delays reaching the health facility for delivery services. Data on participants’ socio-demographics, delay one, delay two, three and determinants of delays one and two and three were collected using questionnaires. We calculated frequencies and proportions for factors contributing to delays as well as Prevalence Odds Ratios (POR) to identify risk factors for the delays.

**Results:**

A total of 614 mothers were interviewed, median age 28 years (range, 14–52 years). The prevalence of Delay One was 23.3% (143/614), and Delay Two was 26.9% (165/614). Mother with secondary education were associaited with delay one (aPOR = 2.3; 95% CI:1.14, 4.46). These was an association between perceived delay-two and previous pregnancy-related complications (aPOR = 1.6; 95% CI:1.071, 2.538) and poor condition of roads (POR = 2.34; 95%CI, 1.15, 4.77). Additinally, there was an association between delay-three and previous-related complication during last pregnancy (aPOR = 1.9; 95% CI: 1.055, 3.67).

**Conclusions:**

This study revealed a high prevalence of perceived delays one and two for mothers to access obstetric services. Delays were mainly related to transport difficulties, low knowledge of pregnancy-related complications, and costly obstetric services. A practical strategy for birth preparedness and readiness to reduce delays is urgently needed.

## 1. Background

Globally, in 2021, the World Health Organization (WHO) and United Nations Population Fund (UNFPA) estimated that 810 women continued to die each day due to complications of pregnancy and childbirth [1]. Nearly 95% of these deaths occurred in low-and-middle income countries and could have been prevented [2]. Globally, the UNFPA reported that one woman died every two minutes from pregnancy or childbirth-related causes, with more than 80% of these deaths occurring in sub-Saharan Africa (SSA) in 2017 [3]. Of the estimated 295,000 maternal deaths that occurred worldwide, 277,300 (94%) occurred in low-resource countries. Maternal deaths in SSA and Southern Asia accounted for 86% (254 000) of the global estimates [4]. Furthermore, SSA alone accounted for 70% (202 000) of maternal deaths, while Southern Asia accounted for nearly one-third (47 000) [5]. The situation is worse in West African countries, where the maternal mortality ratio (MMR) is 679 deaths per 100,000 live births in Guinea, 725 in Liberia, and 717 per 100,000 in Sierra Leone compared with the SSA average of 546 [6, 7]. In Sierra Leone, recent study indicated that maternal mortality rate was 516 per 100, 000 live birth in 2020 [8].

In a systematic analysis of maternal mortality causes conducted in 2023, found that the common direct causes of maternal deaths included postpartum haemorrhage, pregnancy related infections, hypertensive disorders of pregnancy, postpartum infections, obstructed complications (6%), and an unticipated complication of management and pregnancy with the aborted outcome [9].

Low access to appropriately skilled birth attendants at delivery and timely referrals to emergency obstetric care services are the two most critical factors that cause maternal deaths as shown in a study conducted in Ethiopia [10]. However, in Sub-Saharan Africa (SSA), including Sierra Leone, women continue to have insufficient access to skilled birth attendants at delivery and to timely referrals to emergency obstetric care services [11]. Ninety-nine percent of women’s deaths during pregnancy, childbirth, or postpartum in developing countries, particularly in SSA, are due to limited or late access to skilled delivery services [12]. The UNFPA has indicated that maternal deaths in SSA result from inadequate access to timely emergency obstetric services [13].

In Sierra Leone, the factors that contribute to delays in accessing emergency obstetric services have not been studied and remain largely unknown. Thaddeus and Maine classified the factors that lead to delays for pregnant women in receiving appropriate maternal health care which become the relevant factors contributing to maternal death into three phases. The first phase is the delay in deciding to seek care by pregnant women, their husbands, or other decision-makers in their families, the second phase is the delay in reaching the health facility after a decision is made to seek care, and the third phase is the delay in receiving adequate care services after reaching at the health facility [14]. The first and Second Delays are related to either accessibility or factors involving family and the community, such as deciding to seek care and transportation-related challenges. The third Delay is related to challenges in receiving care at health facilities, including the quality of care and other factors within the health facility. An individual woman can experience all three delays during one pregnancy.

Factors that contribute to the First Delay include lack of knowledge about the seriousness of complications, lack of knowledge about where to receive services, requiring permission from family decision-makers, and cultural beliefs that may prevent mother or new born from leaving home. Other factors found in the literature to contribute to the First Delay include high cost of services, negative previous healthcare experiences, educational level, low status of women, perceived low-risk pregnancy, and perceived low quality of care [15]. Factors contributing to the Second Delay include the wide distribution of healthcare facilities, long travel time from clients’ homes to the healthcare facility, unavailability of transportation, high transportation cost, security concerns, and poor condition of roads [16]. Third Delay-related determinants include lack of a referral system and limited or insufficient supplies, equipment, and competent personnel at healthcare facilities [17].

Studies in SSA have revealed that first and second delays for women to access emergency obstetric care for pregnancy and delivery-related complications are significant contributors to maternal death [18, 19]. In Sierra Leone, the 2017 Maternal Death Surveillance and Response (MDSR) report identified delays in the management of pregnancy and delivery-related complications such as pre-eclampsia and post-partum haemorrhage (PPH) to contribute to more than 98% of maternal deaths in the country [20]. The number of studies that have investigated determinants of delays in accessing facility-based obstetric care have either employed qualitative methods or had small sample sizes, making it difficult to generalize their findings. It remains unclear whether factors identified in the literature to cause delays to women accessing obstetric services are relevant to Sierra Leone.

Designing and implementing effective programs to reduce maternal mortality rates requires a deeper understanding of the factors contributing to delays, which are a critical pathway to maternal mortality. Understanding mothers’ perspectives regarding the presence and determinants of delays in accessing facility-based obstetric care services is paramount in developing programs and policies that will respond effectively to reduce delays. This study, therefore, investigated women’s views and perceptions on the presence of delays and the factors that might contribute to delays in accessing facility-based obstetric care in Sierra Leone to inform recommendations to policymakers to mitigate the adverse effects of these factors on maternal deaths in Sierra Leone.

## 2. Materials and methods

### 2.1. Study design

This study was a community-based cross-sectional design. It was conducted in the districts of Western Area Urban, Kenema, Bo, and Bombali in Sierra Leone in June and July 2018.

### 2.2. Study area

This study was conducted in Kenema, Bo, Bombali, and Western Area Urban districts in Sierra Leone. Kenema district is located 307 km to the east of the capital Freetown and is one of the three districts in the Eastern province. There are 45 chiefdoms in the three districts (Kenema, Bo, and Bombali) and eight zones in Western Area Urban. Bo district is located 248 km to the southeast of Freetown and is one of the four districts in the southern province. Bombali district is located 187 km north of Freetown and is one of the five districts in the northern province, while Western Area Urban is located in the western region and is one of the two districts making up Freetown, the capital city of Sierra Leone. At the time of the study, the four districts had a total population of 3,038,314 inhabitants. The four districts had an expected number of annual deliveries of 124,571, which was accounted for 4.1% of the total population. There were 439 health facilities in the four districts: 31 hospitals, 99 community health centers, 106 community health posts, and 203 maternal and child health posts.

In Sierra Leone, there are 1172 healthcare facilities that provide maternal serives in the entire coutry. These facilities are distributed as follows: 684 Maternal and Child Health Post (MCHP), 277 Community Health Center (CHC), 450 Community Health Post (CHP), 222 Clinic, and 88 Hospital. Maternal health services, including ANC, deliveries, and postnatal services, are available inall these health facilities. However, women identified as a high risk during pregnancy are referred to the nearest hospital. The lowest level of healthcare facility is the Maternal and Child Health Post (MCHP), usually located in remote communities and managed by lower cadre of healthcare workers. The Second to the MCHP is the Community Health Post (CHP) which is located in remote communities. The following that is the Community Health Center (CHC), which is located at the chiefdom level and staffed by better-qualified health workers such as community health officers (clinical assistants) and midwives. Hospitals are located at the district level, handle all severe cases and managed by highly qualified health professional such as doctors, registered nurses, surgical community health officers (CHOs), and midwives.

In the districts where this study was condcuted (Bo, Bombali, Kenema, Western Area Rural), there were a total of 478 health facilities providing healthcare services to pregnant women. In Bo District, there were 32 CHC, 40 CHP, 78 MCHP, and 7 hospitals. Bombali District had 13 CHC, 35 CHP, 36 MCHP, and 6 hospitals. Kenema District has 32 CHC, 37 CHP, 36 MCHP, and 5 hospitals. Wesern Arae Rural Distict had 23 CHC, 20 CHP, 17 MCHP, and 34 hospitals.

### 2.3. Study population

The target population included all women who had delivered between May 1, 2017, and June 30, 2018, in the four districts of Sierra Leone (Western Area Urban, Kenema, Bo, and Bombali), regardless of their birth outcome. The study population consist of women who delivered between May 1, 2017, and June 30, 2018, and were residing in the four selected districts at the time of the stud. Study participants were women who fulfilled the selection criteria and consented to participate in the interview.

### 2.4. Inclusion and exclusion criteria

#### 2.4.1. Inclusion criteria

All women who resided in the selected study area for the last 12 months and who gave birth between May 1, 2017, and June 30, 2018, in Sierra Leone regardless of their birth outcomes.

#### 2.4.2. Exclusion criteria

Women who met the inclusion criteria but who were too ill to participate in the survey at the time of data collection were excluded from the study.

### 2.5. Sample size

The Epi.info 7.2 Stat Calc population survey module found at www.cdc.gov/epiinfo was used to calculate the sample size based on the population size of districts, an expected frequency delay of 50%, a margin error of 5%, and a design effect of 1.5. The calculated sample size was 605.

#### 2.5.1. Selection of study districts

The four districts, Western Area Urban, Bombali, Bo, and Kenema, are regional cities for the four administrative provinces of Sierra Leone and were purposively selected. Sierra Leone has four administrative regions, West, North west, South, and East. The chiefdoms or zones (55 in total) in the four selected districts were identified as study clusters, and their respective populations were determined. The sample size contribution from each cluster, chiefdom, or zone, was calculated proportionate to its population size. The sample size contribution by each of the 55 clusters was determined by multiplying the study sample size of 605 by its corresponding proportion of the expected number of deliveries in the last year at the interview date. The starting household for the interview within each selected community was randomly chosen.

### 2.6. Data collection

We developed a semi-structured questionnaire to collect data on socio-demographic variables (such as age, home address etc), perceived delays in accessing health facility-based deliveries, and perceived factors contributing to delays in accessing facility-based obstetric care by pregnant women. The questionnaire was piloted to improve the clarity of questions, internal consistency, and face validity [21]. Field Epidemiology Training Program (FETP) intermediate and frontline participants were trained on the research methods, including selecting the study participants and administering the questionnaire for data collection. Data was collected from study participants through face-to-face interview using an electronic questionnaire developed on the Epi-info version 7 software platform. Data collection was supervised by the FETP resident advisor assisted by mentors, to ensure high quality.

### 2.7. Statistical analysis

The collected data was checked for completeness and consistency at the end of each day. After the data collection exercise, the data was merged and cleaned before analysis using Epi Info 7. Mean, range, and standard deviation for age were calculated and proportions for responses to questions about factors perceived by study participants to cause delays were determined. A logistic regression model was built based on the available data and literature. Univarite analysis was used to identify variables associated with outcome variables. A cut-off point of 0.2 was used as a sigificant level to include variables in the multivariate analysis. In multivariate analysis, prevalence odds ratios (POR) at a 95% confidence interval (CI), for perceived delays by explanatory variables were calculated. A cut-off of 0.05 was used to cosider variables as statistically significant in the multivariate analysis.

### 2.8. Ethical considerations

This study was approved by the Sierra Leone Ethics and Scientific Review Committee (SLESRC), with letter number (Version: 09 April 2018). Written informed consent was obtained from all study participants. Data obtained during the interview was stored on a secure computer, confidentiality of participants was maintained. Written informed consent was also obtained from each participant. For participants under 18 years old, consent was obtained from their parents or guardians.

## 3. Results

### 3.1.Demographic characteristics

Out the 614 mothers interviewed, 78 (12.7%) were adolescents aged 14–19 years, 524 (85.34%) were 20 to 39 years, and 12 (1.9%) were 40 years and older. Most of the mothers interviewed were 25 to 29 years old (29.5%) and 20 to 24 years (26.8%). The median age of the mothers interviewed in this study was 28 years (range, 14–52 years).

Nearly one-third (31.9%) of the mothers had received no education, while half (50.6%) had received secondary education and above. The majority of the mothers (77.0%) were married, and 69.5% of them were Muslims. The main tribes of the mothers were Mende (42.0%), Temne (23.0%), and Limba (12.0%). More than half (57.8%) of the mothers had a permanent source of income, with trading (41.0%) and farming (31.0%), being the primary sources of income.

### 3.2. Obstetric characteristics

In this study, most of mothers (80.3%) reported having had four or fewer pregnancies, with only four (0.7%) being pregnant at the time of the study. Most mothers (85.0%) reported no complications during their last pregnancy and delivery. Out of the 614 mothers who reported complications during their last pregnancy, 43 (43.4%) had reported hemorrhage, 28 (28.3%) had obstructed labor, and 15 (15.2%) had hypertensive disease of pregnancy. During their last delivery, 572 (93.2%) out of 614 mothers delivered at a health facility, while 31 (5.1%) delivered at home. The majority, 605 (98.5%) of the 614 mothers interviewed, stated that a health facility was their preferred place of delivery for their next pregnancy (Table 1).

**Table 1:** Distribution of obstetric characteristics of the study participants, Western Area Urban, Bo, Kenema, Bombali districts, Sierra Leone.

Characteristics	Number (n = 614)	Percentage (%)
**Number of previous pregnancies at the time of the study**		
1	141	22.9
2	147	24.0
3	112	18.2
4	93	15.2
≥5	121	19.7
**Pregnant at the time of the study**		
Yes	4	0.7
No	606	98.7
Don’t know	4	0.6
**Complications in the last pregnancy**		
Yes	91	14.8
No	522	85.0
Don’t know	1	0.2
**Type of complication in the last pregnancy**		
Hemorrhage	43	43.4
Obstructed labor	28	28.3
Hypertensive disease of pregnancy	15	15.2
Sepsis	5	5.1
Retained placenta	4	4.0
Others	4	4.0
**Number of previous deliveries at the time of the study**		
1	147	23.9
2	151	24.6
3	111	18.1
4	92	15.0
≥5	113	18.4
**Place of the last delivery**		
Health facility	572	93.2
Home	31	5.1
On transit	6	1.0
Home of traditional birth attendant	5	0.8
**Preferred place for future delivery**		
Health facility	605	98.5
Home	4	0.7
Home of traditional birth attendant	5	0.8

### 3.3. The prevalence of perceived delays in deciding to seek health facility delivery services, reaching a place of delivery and accessing health facility services

The overall prevalence of perceived delay in deciding to seek health facility delivery services, Delay One, is 23.3%, and the overall prevalence of perceived delay in reaching a place of delivery, Delay Two, is 26.9%. Bo district has the highest prevalence of perceived delay in deciding to seek health facility delivery services, Delay One, (45.2%), and perceived delay in reaching a place of delivery, Delay Two, (26.8%), followed by Bombali district with 21.8% perceived delay in deciding, Delay One, and 31.6% perceived delay in reaching a place of delivery, Delay Two, the overall delay in accessing the health facility services, Delay three was 90%.

### 3.4. Factors contributing to the perceived delay in deciding to seek health facility delivery services, delay one

Between 54.9% and 58.1% of the study participants either agreed or strongly agreed with the statements that the low socio-economic status of the women (357, 58.1%), lack of money to pay for high delivery-associated costs (345, 56.2%), low knowledge about complications during pregnancy (345, 56.2%), lack of access to funds in case of complications 345, 56.2%), and long distance to the health facility (337, 54.9%) are significant contributors to Delay One. On the other hand, 50.2% to 62.9% of the study participants interviewed either disagreed or strongly disagreed with the statements that the need for permission from the family decision maker (338, 55.1%), perceived low quality of care (338, 55.1%), low educational level of a pregnant woman (326, 53.1%), previous bad experience with family staff in the health facility (356, 58.0%), negative or unsupportive cultural beliefs (377, 61.4%), and lack of knowledge on where to receive delivery services (387, 63.0%) contribute to Delay One. Almost equal numbers of the study participants interviewed either agreed or strongly agreed or disagreed, or strongly disagreed that the high cost of transport to the health facility (295, 48.0%) and the low perceptions of risk factors (299, 48.7% and 256, 41.7% respectively) contribute to Delay One (Table 2).

**Table 2:** Factors perceived by study participants to contribute to Delay One in Bo, Kenema, Bombali, and Western Area Urban districts, Sierra Leone, 2018.

Factors	Strongly agree (%)	Agree (%)	Strongly agree + Agree (%)	Undecided (%)	Disagree (%)	Strongly disagree (%)	Disagree + strongly disagree (%)
Low socio-economic status of the women	28.0	30.1	58.1	6.4	22.6	12.9	35.5
Lack of money to pay for delivery-associated costs	31.8	24.4	56.2	5.1	23.9	14.8	38.8
High cost of delivery services	29.6	26.6	56.2	5.7	23.8	14.3	38.1
Low knowledge about complications during pregnancy	27.4	28.8	56.2	4.7	24.8	14.3	39.1
Lack of access to funds in case of complications	27.4	28.8	56.2	4.4	21.8	17.6	39.4
Long distance to the health facility	28.8	26.1	54.9	2.0	25.2	17.9	43.2
High cost of transport to the health facility	21.7	27.0	48.7	3.4	27.9	20.0	47.9
Low perceptions of risk factors	19.4	29.3	48.7	9.6	27.4	14.3	41.7
Need for permission from family decision maker	17.4	23.6	41.1	3.9	33.9	21.2	55.1
Perceived low quality of care	14.0	25.6	39.6	7.7	36.2	14.0	50.2
Low educational level of pregnant mother	14.8	24.4	39.3	7.7	33.2	19.9	53.1
Previous bad experience with the staff in the health facility	14.7	21.2	35.8	6.2	35.99	22.0	58.0
Negative or unsupportive cultural beliefs	15.2	17.3	32.4	6.2	38.6	22.8	61.4
Lack of knowledge on where to receive delivery services	15.2	16.1	31.3	5.9	36.64	26.2	62.9

### 3.5. Factors contributing to the perceived delay in reaching the health facility for delivery, Delay Two

Almost equal numbers of study participants interviewed either agreed or strongly agreed that the high transport cost contributed to Delay Two (47.9%), while 48.7% disagreed or strongly disagreed. Slightly over fifty percent of the study participants (51.8%) strongly agreed or agreed that lack of transport to a health facility contributed to delay in reaching the health facility for delivery services, Delay Two. Again, over fifty percent of the study participants, 54.9%, strongly agreed or agreed that the long distance from the mother’s home to the health facility contributed to Delay Two. Between 53.6% to 57.5% of the study participants disagreed or strongly disagreed that poor road conditions (329, 53,6%) and poor security concerns (353, 57.5%) contributed to Delay Two (Table 3).

**Table 3:** Factors perceived by study participants to contribute to’ Delay Two in Bo, Kenema, Bombali, and Western Area Urban districts, Sierra Leone, 2018.

Factors	Strongly agree, n (%)	Agree, n (%)	Strongly agree + Agree	Undecided, n (%)	Disagree, n (%)	Strongly disagree, n (%)	Disagree + Strongly disagree
Long distance to the health facility	28.8	26.1	54.9	2.0	25.2	17.9	43.2
Lack of transport to the health facility	24.8	27.0	51.8	2.0	26.0	20.4	46.3
High cost of transport to the health facility	21.7	27.0	48.7	3.4	27.9	20.0	47.9
Poor condition of roads	18.6	24.6	43.2	3.3	30.6	23.0	53.6
Poor security concerns	12.7	19.2	31.9	10.6	34.5	23.0	57.5

### 3.6. Factors contributing to the perceived delay in receiving health facility delivery services by mothers, Delay Three

More than half of the study participants (56.2%) agreed or strongly agreed that the lack of medicines contributed to delays in receiving health facility delivery services, Delay Three. Also, 59.6% of the study participants agreed or strongly agreed that the high cost of delivery services contributed to Delay Three. On the contrary, 53.4% of the study participants disagreed or strongly disagreed that a lack of skilled staff contributed to Delay Three. Simialry, 54.2% of the study participants disagreed or strongly disagreed that the poor attitude of the staff contributed to Delay Three (Table 4).

**Table 4:** Factors perceived by study participants to contribute to Delay Three in Bo, Kenema, Bombali, and Western Area Urban districts, Sierra Leone, 2018.

Factors	Strongly agree	Agree	Strongly agree + agree	Undecided	Disagree	Strongly disagree	Disagree + strongly disagree
Lack of medicines	27.2	32.4	59.6	5.2	23.1	12.1	35.2
High cost of delivery services	29.6	26.6	56.2	5.7	23.8	14.3	38.1
Delay in accessing ambulance services	24.1	23.5	47.6	7.5	26.6	18.4	45.0
Poor attitude of staff	17.6	22.3	39.9	5.9	34.0	20.2	54.2
Lack of skilled staff	20.9	17.4	38.3	8.3	29.2	24.3	53.4
Delay in being referred	16.1	19.1	35.2	16.6	30.8	17.4	48.2

### 3.7. Perceived delay in deciding to seek health facility delivery services and perceived delay in reaching health facility by socio-demographic- and obstetric-characteristics of mothers

Study participants in the age group (25–29 years) reported the highest perceived delays in deciding to seek delivery services, with 48 out of 143 (33.6%), which was followed by the age group (20–24 years) with 33 of 143 (23.1%). Among the 143 study participants who reported delay in deciding to seek health facility delivery services, Delay One, those who were married reported the highest perceived Delay One 111 (77.6%) than those who were single, separated, or cohabiting. Fifty of the one hundred and forty-three study participants (35.0%) who reported a delay in deciding to seek health care delivery services, Delay One, had no education. Study participants within the 25–29 year age group reported the highest perceived delay in reaching the place of delivery, Delay Two, 54 (32.7%), followed by the 20–24 years age group with 40 (24.2%). Study participants who were married reported more perceived Delay Two, at129 (78.2%).

#### 3.7.1. Bivariate analysis

There was no statistically significant association between study participants who reported having a permanent source of income and perceived delay in deciding to seek health care delivery services, Delay one (POR = 1.38; 95% CI: 0.84, 2.28). The odds of havig Delay one was 2.3 time among mothers with secondary education as compared to those without education (POR = 2.3; 95% CI: 1.14, 4.46) (Table 5). Also, no statistically significant association was observed between study participants who reported having a permanent source of income and perceived delay in reaching health facilities, Delay Two (POR = 1.18; 95% CI: 0.80,1.72) (Table 6). However, there was a statistically significant association between study participants who reported having had one or more complications in the previous pregnancy and the perceived delay in reaching a health facility, delay two (POR = 1.80; 95% CI: 1.13, 2.83) (Table 6).

**Table 5:** Study participants’ perceived Delays One by demographic- and obstetric-characteristics, in Sierra Leone, 2018.

Characteristic	Delay in deciding (Delay One)		Crude POR (95% CI)	Adjusted POR (95% CI)
	Yes, n (%)	No, n (%)		
**Age-group (years)**				
14–19	27(35)	51 (65)	2.6 (0.54, 12.96)	
20–24	33(20)	132 (80)	1.3 (0.26, 5.98)	
25–29	48(27)	133 (73)	1.8 (0.38, 8.53)	
30–34	18(20)	91 (80)	0.9 (0.20, 4.90)	
35–39	15(22)	54 (78)	1.4 (0.27, 7.03)	
≥ 40	2(17)	10 (83)	Ref	
**Marital status**				
Single	25 (25)	77(75)	1.4 (0.52, 3.81)	
Married	111 (24)	361(76)	1.3 (0. 53, 3.32)	
Separated	1 (20)	4(80)	1.1 (0.10, 11.52)	
Co-habiting	6 (19)	26(18)	Ref	
**Educational level**				
No education	50 (26)	146 (74)	Ref	Ref
Primary	30 (28)	77 (72)	1.1 (0.67, 1.93)	1.3 (0.79–2.083)
Secondary	51 (20)	209 (80)	0.7 (0.46, 1.11)	**2.3 (1.14–4.46)**
Tertiary institution	12 (24)	39 (76)	0.9 (0.44, 1.85)	1.1 (0.508–2.41)
**Permanent source of income**				
Yes	87(25)	268(75)	1.2 (0.80,1.72)	
No	56(22)	203(78)	Ref	
**Number of pregnancies at the time of study**				
1	37(26)	104(74)	1.3 (0.73, 2.31)	
2	31(21)	116(79)	0.9 (0.54, 1.76)	
3	26(23)	86(77)	1.1 (0.60, 2.05)	
4	23(26)	70(74)	1.2 (0.63, 2.28)	
≥5	26(21)	95 (79)	Ref	
**Complications in the last pregnancy**				
Yes	26 (29)	65 (71)	1.4 (0.84, 2.28)	
No	117 (22)	405 (78)	Ref	

**Table 6:** Study participants’ perceived Delays Two by demographic- and obstetric-characteristics, in Sierra Leone.

Characteristic	Delay in reaching (Delay Two)		Crude POR (95% CI)	Adjusted POR (95% CI)
	Yes, n (%)	No, n (%)		
**Age-group (years)**				
14–19	27(35)	51 (56)	5.8 (0.71, 47.36)	
20–24	40(24)	125 (76)	3.5 (0.44, 28.01)	
25–29	54(30)	127(70)	4.6 (0.59, 36.99)	
30–34	24(22)	85 (78)	3.1 (0.38, 5.18)	
35–39	19(28)	50 (72)	4.2 (0.50, 34.49)	
≥ 40	1(8)	11 (92)	Ref	
**Marital status**				
Single	28 (27)	74(73)	1.6 (0.61, 4.40)	
Married	129(27)	343(73)	1.6 (0.66, 4.04)	
Co-habiting	2 (40)	3 (60)	2.9 (0.39, 21.28)	
Separated	6 (19)	26(81)	Ref	
**Educational level**				
No education	56 (29)	140 (71)	Ref	
Primary	38 (36)	69 (64)	1.4 (0.84, 2.28)	
Secondary	57 (22)	203 (78)	0.7 (0.46, 1.08)	
Tertiary institution	14 (27)	37 (73)	0.9 (0.48, 1.88)	
**Permanent source of income**				
Yes	90(25)	265(75)	0.8 (0.58, 1.19)	
No	75(26)	184(74)	Ref	
**Number of pregnancies at the time of study**				
1	41(29)	100(71)	1.9 (0.57, 1.65)	
2	34(23)	113(77)	0.7 (0.41, 1.23)	
3	32(29)	80(71)	0.9 (0.54, 1.66)	
4	22(24)	71(76)	0.7 (0.39, 1.36)	
≥5	36(30)	85(70)	Ref	
**Complications in the last pregnancy**				
Yes	34 (37)	57 (63)	1.8 (1.13, 2.87)	**1.6 (1.071–2.538)**
No	130 (25)	392 (75)	Ref	Ref

The odds of having Delay three was 0.13 times among single mother as compared to sperrated mother (POR = 0.13; 95% CI: 0.029, 0.60). The odds of having Delay three was 0.10 times among married mother as compare to sepearted mother (POR = 0.10; 95% CI: 0.024, 0,41). The odds of havig Delay three was 0.03 among co-habitat mothers as compared to sepeparted mothers (POR = 1.80; 95% CI: 0.002,0.36) (Table 7).

**Table 7:** Study participants’ perceived Delays Three by demographic- and obstetric-characteristics, in Sierra Leone, 2018.

Characteristic	Delay in reaching (Delay Two)		Crude POR (95% CI)	Adjusted POR (95% CI)
	Yes, n (%)	No, n (%)		
**Age-group (years)**				
14–19	27(35)	51 (56)	O.9 (0.100, 8.35)	
20–24	40(24)	125 (76)	1.1 (0.143, 9.75)	
25–29	54(30)	127(70)	1.2 (0.157, 10.55)	
30–34	24(22)	85 (78)	1.3 (0.161, 11.48)	
35–39	19(28)	50 (72)	1.0 (0.114, 9.56)	
≥ 40	1(8)	11 (92)	Ref	
**Marital status**				
Single	28 (27)	74(73)	0.13 (0.029, 0.60)	**0.14 (0.029, 0.60)**
Married	129(27)	343(73)	0.10 (0.024, 0,41)	**0.11 (0.024, 0,41)**
Co-habiting	2 (40)	3 (60)	0.03(0.002,0.36)	**0.03 (0.002,0.36)**
Separated	6 (19)	26(81)	Ref	Ref
**Educational level**				
Primary	38 (36)	69 (64)	0.4 (0.412, 1.05)	
Secondary	57 (22)	203 (78)	0.9 (0.504, 1.66)	
Tertiary institution	14 (27)	37 (73)	1.1 (0.403, 2.75)	
No education	56 (29)	140 (71)	Ref	
**Permanent source of income**				
Yes	90(25)	265(75)	1.2 (0.744, 2.24)	
No	75(26)	184(74)	Ref	
**Number of pregnancies at the time of study**				
1	41(29)	100(71)	0.9 (0.41, 1.90)	
2	34(23)	113(77)	1.0 (0.45, 2.25)	
3	32(29)	80(71)	0.9 (0.37, 2.15)	
4	22(24)	71(76)	0.7 (0.32, 1.75)	
≥5	36(30)	85(70)	Ref	
**Complications in the last pregnancy**				
Yes	34 (37)	57 (63)	1.9 (1.101, 3.61)	**1.9 (1.055–3.67)**
No	130 (25)	392 (75)	Ref	Ref

#### 3.7.2. Multivariate analysis

In multivariate analysis only one factor had an association with delay one. The odds of having delay one was 2.3 times higher among women with secondary school education as compared to those without formal education (aPOR = 2.3; 95% CI:1.14–4.46) (Table 5). Similairly, one variable was associated with delay two. The odds of getting complecation in the last preganacy was 1.6 time among women who perceived delay two and it was statiscally significan (aPOR = 1.6; 95% CI:1.071, 2.538) (Table 6). The odds of receiving health facility delivery services was 0.14 times among single womant (aPOR = 0.14; 95% CI: 0.029, 0.60), 0.11 times among married women (aPOR = 0.11; 95% CI: 0.024, 0,41), and 0.03 times among cohipiting women (aPOR = 0.03; 95% CI: 0.002,0.36), and the difference is statistically significant. Additionally, the odds of women who exereinced complication during the last pregnancy was 1.9 times to exereince delay three as compared to those who did not have complications (aPOR = 1.9; 95% CI: 1.055–3.67) and it was statistically significant.

## 4. Discussion

This study investigated women’s views and perceptions on delays in accessing facility-based obstetric care in Sierra Leone, specifically focussing on Delays One Two and Three, aw well as the factors contributing to these delays using the Thaddeus and Maine three-delay model, as the guiding framework [22]. This study findings indicated a high prevalence of perceived delay in deciding to seek health facility delivery services (Delay One), delay in reaching a place of delivery (Delay Two) and delay in accessing maternal services at health facility (Delay Three). Our study found a higher prevalence of Delays One, Two and Three compared to the findings in Bangladesh [23]. However, the prevalence of Delays One and Two in our study was lower than in studies conducted in Ethiopia (46.8%) and Malawi (39,4%) for Delay One and 44.0% (Ethiopia) and 59.6% (Malawi) for Delay Two [17, 24].

Hemorrhage had the highest proportion of pregnancy complications reported by mothers in their last pregnancy. Hemorrhage cold be the cause of death among mothers during delivery. A study conducted by the WHO reported that heamorrhage is a leading cause of maternal death [25]. Studies in SSA have also identified bleeding occurring after delivery (postpartum haemorrhage (PPH), caused by retained products and mismanagement of the third stage of labour, as a number one cause of maternal mortality [26, 27].

This study found that only 5% of the mothers had delivered at home. The reasons for delivering at home could be the same reasons identified by the current study to contribute to Delays One and Two: high cost attached to delivery servicesand the negative attitude of health workers in the health facilities which might contributed in Delay Three [22]. Similar findings on home delivay was reported in the first annual MDSR report in Sierra Leone in 2017 [28].

This study has identified the low socio-economic status of women, lack of fund money to cover delivery-associated costs, high cost of delivery services, insufficient knowledge about complications during pregnancy, lack of access to funds in case of preganacy complications, and long distance to health facilities as contributors to the first delay in Sierra Leone. This finding is consistant with a case series study of maternal deaths in Nepal that showed that women in poor and marginalized communities are at risk of death due to first delay [29]. A study conducted in Zimbabwe also reported similar findings [30]. Despite the implementation free health care for pregnant and lactating mothers in Sierra Leone in 2010, this current study found that high delivery-associated costs still significantly contribute to Delays One, Two and Three. These findings suggest that pregnant women are being illegally charged for maternal services at public health facilities for maternal services,leading financial challenges for many of the women interviewed.

Similar to the study in India, which found that the second major contributing factor to maternal deaths was the delay in reaching first level health facility, [31] the current study identified long distance and lack of transport to a health facility as the main contributors to Delay Two. A study in Bangladesh also found that long distance to health facilities and the high cost of transport were the two main contributors to Delay Two [23]. Long distances can affect womens’ decision to seek health facility delivery services, a finding supported by numerous studies. For example, a study conducted in Sudan found that women were unwilling to spend more than 30 minutes traveling time to health facilities [32]. Our study also identified the lack of medicines and the high cost of delivery services as the main contributors to delays in receiving health facility delivery services, Delay Three. The lack of commodities which is often associated with poor quality care services in health facilities negatively influence mothers’ decision to seek health facility-based maternal services, leading them to prefer delivering at home [33].

Based on our findings, mothers who had secondary education were delayed in seeking health facility delivery services. This could be attributed to the negative attitudes this type of mothers have towards health facilitiy delivery. A study conducted in Ethiopia found that mothers’ negative attitudes toward institutional delivery was associated with a delay in seeking care at health facilities [34]. Although the current study did not find an association between age groups, marital status, source of income and Delays One and Two, results from a study conducted in South Africa showed an association between socio-demographic characteristics of mothers and Delays One and Two [35].

This study found that mothers with previous pregnancy complications had a significant association with perceived Delay Two. This could be due to the negative experience that they had druing their previous delivery in the health facility. Astudy conducted in Ethiopia found that previous pregnancy problems was associated with delay in accessing health facility delivery [36]. Our findngs indicated that separated mothers were more likely to experience Delay Three. This could be likely that separated mothers do not have the finical capacity to pay for the healthcare services if they are not employed or having someone to take care oh them. Study conducted in Ethiopia found that unemployed mothers was associated with the delay in seeking care at health facilities [34]. Another study conducted in the Oromia region of Ethiopia reported low monthly income was associated with maternal delay in accessing emergency obstertric care for mother receiving insititutional delivery services [37].

### 4.1. Study limitation and sterntghs

The study design employed was a cross-sectional study, which could not a establish causal association between independent and outcome variables. For delay one, two and three we combined agree and strongly agree in one group as well as for disagree and strongly disagree. Those who were neutral were included in these groups which might cause bias. To overcome the study limitation, households were randomly selected to reduce any potential selection bias. Also, the study questionnaire was piloted to improve the quality of data collection and reduce recall bias. The strength of this study was that it provided information on delays in accessing the health facility-based maternal delivery services in Sierra Leone, which can inform policymakers to reduce and prevent maternal mortality in the country.

## 5. Conclusions

We found a high prevalence of perceived Delays one, two, and three for mothers to access obstetric services. Secondary education of mothers seeking delivery at the health facility was associated with delay one. Previous pregnancy complications at least one time was associated with Delays two and three.

### 5.1. Recommendations

1. We recommend to develop comprehensive strategy for birth preparedness and complication readiness to reduce all of the three types of delays is urgently needed.
2. We recommend that they develop a comprehensive strategy of “birth preparedness and complication readiness” to reduce all Three Delays
3. Policy makers to develop interventions that enhance women’s socioeconomic status such as Income generating activities
4. We recommend Policy makers to improve and maintain effective and efficient ambulance referral services throughout the country
5. We recommend to sensitize the community and women on the pregnancy-related risk factors and complications
6. We recommend qualitative studies to understand why mothers who reported one or more complications reported more perceived Delay Two than those who did not report such complications.

## Supporting information

S1 Checklist Human participants research checklist. (DOCX)

## Abbreviations

AIDS: Acquired Immunodeficiency Syndrome
CHC: Community Health Center
CHP: Community Health Post
CI: Confidence Interval
FETP: Field Epidemiology Training Program
HF: Health facility
HIV: Human Immunodeficiency Virus
MCHP: Maternal and Child Health Post
MDSR: Maternal Death Surveillance and Response
MMR: Maternal Mortality Ratio
POR: Prevalence Odds Ratios
PPH: Post-Partum Haemorrhage
SLESRC: Sierra Leone Ethics and Scientific Review Committee
SSA: Sub-Saharan Africa
UNFPA: United Nations Population Fund
WHO: World Health Organization
